# Constitutively active *Arabidopsis* cryptochrome two alleles identified using yeast selection and deep mutational scanning

**DOI:** 10.1016/j.jbc.2025.110265

**Published:** 2025-05-21

**Authors:** Amir Taslimi, Axel Jeibmann, Lukas Goett-Zink, Tilman Kottke, Chandra L. Tucker

**Affiliations:** 1 Department of Pharmacology, University of Colorado School of Medicine, Aurora, Colorado, USA; 2 Biophysical Chemistry and Diagnostics, Medical School OWL, Bielefeld University, Bielefeld, Germany; 3 Biophysical Chemistry and Diagnostics, Department of Chemistry, Bielefeld University, Bielefeld, Germany

**Keywords:** *Arabidopsis thaliana*, cryptochrome, flavoprotein, oligomerization, optogenetics, photoreceptor, plant, ultraviolet-visible spectroscopy (UV-vis spectroscopy), yeast two-hybrid

## Abstract

The *Arabidopsis* blue light photoreceptor cryptochrome 2 (CRY2) responds to blue light to initiate a variety of plant light-based behaviors and has been widely used for optogenetic engineering. Despite these important biological functions, the precise photoactivation mechanism of CRY2 remains incompletely understood. In light, CRY2 undergoes tetramerization and binds to partner proteins, including the transcription factor CIB1. Here we used yeast-two hybrid screening and deep mutational scanning to identify CRY2 amino acid changes that result in constitutive interaction with CIB1 in dark. The majority of CRY2 variants show constitutive CIB1 interaction mapped to two regions, one near the FAD chromophore and a second region located near the ATP binding site. Further testing of CRY2 variants from each region revealed three mapping near to the FAD binding pocket (D393S, D393A, and M378R) that also form constitutive CRY2-CRY2 homomers in dark, suggesting they adopt global conformational changes that mimic the photoactive state. Characterization of D393S in the homolog pCRY from *Chlamydomonas reinhardtii* using time-resolved UV–vis spectroscopy revealed that the FAD chromophore fails to form the neutral radical as signaling state upon illumination. Size exclusion chromatography of D393S shows the presence of homomers instead of a monomer in the dark, providing support for a hyperactive variant decoupled from the FAD. Our work provides new insight into photoactivation mechanisms of plant cryptochromes relevant for physiology and optogenetic application by revealing and localizing distinct activation pathways for light-driven CRY2–CIB1 and CRY2–CRY2 interactions.

Plant cryptochromes are blue light photoreceptors that regulate plant growth and a variety of light-cued metabolic and developmental processes. In *Arabidopsis thaliana*, cryptochrome 1 (CRY1) and cryptochrome 2 (CRY2) mediate blue light responses relevant to seedling de-etiolation, seed germination, shade avoidance, and the regulation of flowering time (1, 2, 3). In addition to playing an important role in plant photosensory signaling, CRY2 has been extensively utilized for optogenetic engineering (4, 5). CRY2 exists as a monomer in the dark but undergoes homo-oligomerization with light, forming a tetramer, where it also interacts with partner proteins including COP1 and the transcription factor CRYPTOCHROME-INTERACTING BASIC-HELIX-LOOP-HELIX 1 (CIB1) (6, 7). The CRY2/CIB1 interaction has been utilized for light-dependent induction of protein–protein interactions (4).

CRY1 and CRY2 have similar structural organization comprising two main domains: a conserved N-terminal photolyase homology region (PHR, residues 1–498 of CRY2) that binds the flavin adenine dinucleotide (FAD) chromophore responsible for light detection, and a more divergent and unstructured C-terminal extension (CCE) that has an important role in blue light signaling (8, 9). The PHR domain of all plant cryptochromes binds the FAD in the oxidized state in the dark, then undergoes photoreduction to the FAD neutral radical (FADH^•^) state, representing the active lit state, upon exposure to blue light (10, 11, 12). Coincident with the FAD oxidation state changes, structural changes occur in both the PHR and the CCE domains to induce the biologically active conformation (13, 14, 15, 16). The generally accepted mechanism of phototransduction involves electron transfer from tryptophan residues and a proton transfer from D393 to the FAD, which leads to structural rearrangements in the photoreceptor (11, 15). Binding of ATP to plant cryptochromes *in vitro* and cells strongly stabilizes the active lit state (17, 18, 19, 20). However, the precise conformational changes leading to the biologically active lit state of CRY2, particularly with respect to its CCE domain, remain elusive.

Characterization of constitutively active mutations has provided important insights into the photocycle and signaling mechanisms of photoreceptor proteins (21, 22, 23, 24). To gain further insight into the signaling mechanism of cryptochromes, we used deep mutational scanning combined with a yeast-based selection screen to identify mutations in CRY2 that result in constitutive activity. Using a truncated CRY2 (1- 535) variant with a short CCE, we screened for CRY2 mutants that show enhanced interaction with CIB1 in the absence of blue light, then tested a selected set of these for constitutive homo-oligomeric CRY2-CRY2 interaction. We identified a cluster of variants that show light-independent CIB1 interaction, and three variants that exhibit both constitutive CRY2-CIB1 and CRY2-CRY2 interaction, indicative of a structural change that mimics the CRY2 photoactive state. Characterization of one of these variants, D393S, in the homologous cryptochrome pCRY using time-resolved UV-vis spectroscopy and size exclusion chromatography revealed constitutive self-association in the dark and therefore uncoupling of flavin state and active protein structure. Our work provides further insight into mechanisms and structural changes underlying photoactivation of *Arabidopsis* cryptochrome 2 and highlights the utility of deep mutational scanning methods for characterizing photoactive proteins.

## Results

To gain a better understanding of photoactivation mechanisms of *Arabidopsis* CRY2 and to identify constitutively active variants that could serve as controls in optogenetic studies, we undertook a screen for CRY2 variants that showed enhanced light-independent interaction with the CRY2 binding partner CIB1. We previously had shown by yeast two-hybrid experiments that a truncated version of CRY2, CRY2(535) (containing residues 1–535), interacts with CIB1 as well as itself (CRY2(535)), but only when illuminated with blue light for the duration of the experiment (25). We reasoned that CRY2(535) variants that interact in the dark with both partners, CIB1 and CRY2, would be good candidates for constitutive photocycle mutants. Our strategy involved a primary screen in the dark for CRY2-CIB1 interactors, then a secondary test for light-independent CRY2-CRY2 interaction.

**Figure 1:**
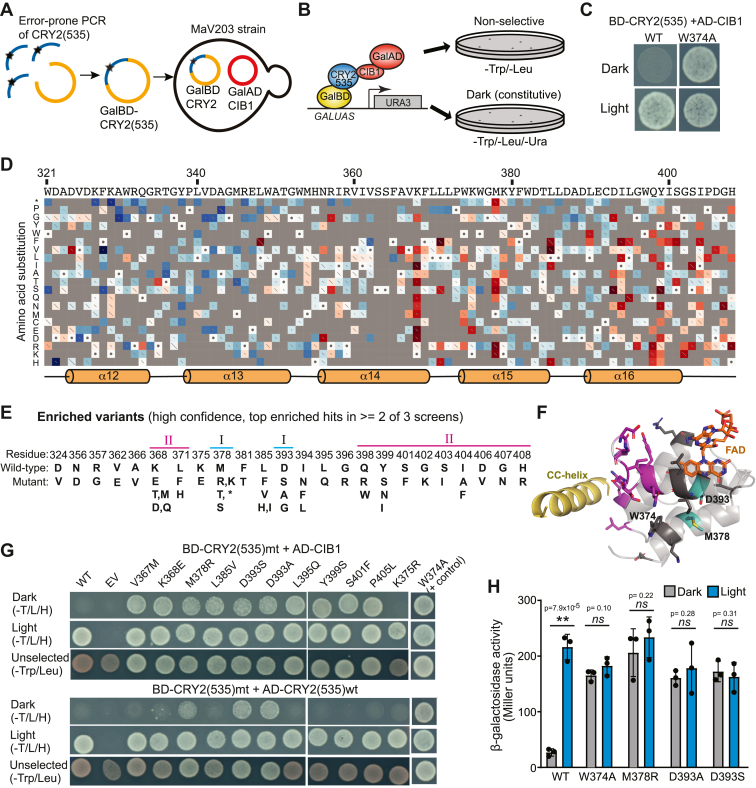
**Mutagenesis and screening for constitutive CRY2-CIB1 interacting mutants.***A*, schematic showing MaV203 yeast strain and constructs used in two-hybrid screen. *B*, schematic indicating the growth selection assay used for screening. Interaction between Gal4BD-CRY2(535) and Gal4AD-CIB1 reconstitutes the Gal4 transcription factor, resulting in the activation of a URA3 reporter and allowing growth on -Ura plates. *C*, MaV203 yeast expressing GAL4BD-CRY2(535) (wt or W374A control) and Gal4AD-CIB1 plasmids. Yeast expressing BD-CRY2(535) wt and AD-CIB1 show light-dependent growth. *D*, schematic showing variants enriched (*red*) or depleted (*blue*) on -Ura plates (data compiled from all three screens). Wild-type CRY2 sequence indicated at *top*, with secondary structure below. The amino acid substitution is indicated at *left*. Wild-type residues are indicated with a *dot* in a *white box*. The length of the slash in the box represents the standard error of the scoring (with a long slash representing greater error). Schematic generated using Enrich2 software (43). *E*, variants showing high enrichment (top 50 enriched in at least two of three screens). Wild-type residue is indicated at *top*, with enriched variant(s) listed below. *F*, CRY2 structure (PDB entry 6K8I) of the screened region (aa 321–412) showing the FAD (*orange*) and side chains of highly enriched residues noted in (*E*) including the positive control W374. Mutants mapping to the surface are classified as Region II (*magenta*) and mutants close to the FAD binding site as Region I (*cyan*). The adjacent CC-helix at the C-terminus of the PHR domain is shown in *yellow*. *G*, yeast two-hybrid interaction results showing wildtype (wt) or indicated variants in GalBD-CRY2(535) tested for interaction with GalAD-CIB1 or GalAD-CRY2(wt) in dark or light on -Trp/-Leu/-His +3mM 3AT plates (-T/L/H). Unselected -Trp/-Leu plates serve as controls. *H*, beta-galactosidase reporter activity resulting from CRY2 mutant-mutant interactions. Indicated variants were placed in GalBD-CRY2(535) and GalAD-CRY2(535) and tested for interaction in strain AH109. All tested variants (other than wild type) showed constitutive activity in dark and a lack of light response. Data shows mean and error (s.d.), three independent experiments. ns, not significant (*p* > 0.05); ∗∗, *p* = 7.9 × 10^−5^, two-tailed unpaired *t* test.

To simultaneously probe the impact of a large variety of structural changes, we utilized a deep mutational scanning approach (26), combining large-scale mutagenesis with deep sequencing to identify enriched and depleted variants. We used an Illumina paired-end sequencing platform that allows sequencing of up to ∼300 base pairs, requiring us to limit the region mutagenized to 300 bp. We targeted amino acids 321 to 412 of CRY2, as this region contains residues that surround the FAD chromophore. We subjected this region to mutagenesis using error-prone PCR, aiming for 1 to 3 mutations within each DNA fragment for an average of 1.9 mutations in each 300 bp region. The pool of mutagenized DNA fragments was incorporated by yeast homologous recombination into a GalBD-CRY2(1–535) plasmid (pDBTrp) in MaV203 yeast already expressing GalAD-CIB1 (pGADT7rec-CIB1), yielding an initial library titer of 500,000 unique recombinants (Fig. 1*A*).

In MaV203 yeast, interaction between CRY2 and CIB1 reconstitutes the Gal4 transcription factor and induces expression of a GalUAS-URA3 reporter, allowing growth on plates lacking uracil (-Ura plates). Transformed yeast were first plated on SC -Trp/-Leu plates, allowing selection for both the GalBD-CRY2(1–535) mutagenic library and GalAD-CIB1 plasmids. To enrich for variants that could interact with CIB1 in the dark, yeast were plated on media lacking uracil (SC -Trp/-Leu/-Ura) and incubated in the dark (Fig. 1*B*). Under these conditions, control yeast expressing wild-type GalBD-CRY2(1–535) and GalAD-CIB1 showed no growth in the dark, despite exhibiting robust growth in blue light (Fig. 1*C*). As a positive control for the assay conditions, we used W374A, which was previously demonstrated to show a constitutive photomorphogenic phenotype and constitutively interact with CIB1 (27, 28, 29). The CRY2 W374A variant showed robust growth on selective media in dark and light (Fig. 1*C*). For the screen, dark/-Ura selected yeast were pooled, then sequenced (Illumina NovaSeq X), along with an unselected pool from the initial (SC -Trp/-Leu) plating that was used to determine the baseline representation of mutant variants prior to selection. We carried out three independent selection experiments: one with a post-selection timepoint at 48 h, and two that underwent two sequential rounds of selection: pooling/replating yeast colonies on fresh plates at 48 h, followed by sequencing at 96 h. Sequencing data were analyzed using the Enrich2 computational tool to generate enrichment scores and standard error. Compiled data from the three screens are shown in Figure 1*D*, with enriched variants in red and depleted variants in blue.

We classified high-confidence enriched variants as those scoring in the top 50 in at least two of the three screens (Fig. 1*E* and Table S1), after removing low-confidence scores (high standard error). Many of the dark-enriched variants mapped to two main regions on the CRY2 structure: Region I, in residues adjacent to the FAD isoalloxazine ring (M378 and D393), and Region II, mapping to an area on the surface of CRY2 containing residues 366 to 369 of α14, and 398 to 408 of α16 and the C-terminal loop region (Fig. 1*F*). Among the high-confidence dark-enriched variants were previously characterized hyperactive CRY2 variants D393G, S401F, and I404F, supporting the validity of the screening process (30, 31). Also supporting the validity of the screen was the fact that nearly all substitutions to stop codons were deleterious.

To confirm the primary screen results, we selected eight high-confidence variants (K368E, M378R, L385V, D393S, D393A, L395Q, Y399S, and S401F) to generate, allowing us to independently confirm the high-throughput sequencing results. The D393S and D393A variants were chosen due to their location at a conserved Asp that acts as proton donor to FAD and is critical for phototransduction (14, 15). In each of these cases, the substituted residues cannot donate a proton to FAD anymore, similar to D393C characterized previously (32). Accordingly, they should interfere with formation of FADH^•^ and stabilization of the active lit state of CRY2, but formation of a constitutive phenotype would not have been expected and motivated a more thorough characterization. In addition to these high-confidence enriched variants, we also generated several controls for testing: a non-enriched control expected to behave similar to wild-type (K375R, which showed a neutral average screen score), and a positive constitutive control (the previously characterized constitutive W374A variant) (27). To explore the validity of other enriched hits that scored below our initial selection criteria, we also tested two variants, P405L and V367M, that showed enrichment in all three screens but were not in the top 50 in two of three screens (Table S1). CRY2 V367M is a natural variant found in the Cvi-0 (Cape Verde Islands) accession, which had previously been characterized as a hyperactive allele associated with early flowering time and photoperiod insensitivity (31, 33).

These 12 mutations were each generated *de novo* in the GalBD-CRY2(535) vector by PCR and homologous recombination in yeast, then the resulting variants were tested for interaction with CIB1 using yeast two-hybrid (Fig. 1*G*, top three rows). In addition, we checked for homomer formation by resolving the interaction with wild-type CRY2 using the same assay (Fig. 1*G*, bottom three rows). To simplify testing, instead of the MaV203 yeast strain used for the initial high-throughput screen, we used AH109 and Y187 two-hybrid reporter strains, which contain a GalUAS-HIS3 reporter and are compatible with a mating-based two-hybrid interaction test. GalBD-CRY2 fusion constructs were expressed in strain AH109 then mated to strain Y187 expressing one of three GalAD plasmids: pGADT7rec (empty vector control), pGADT7rec-CIB1 (expressing GalAD-CIB1), or pGADT7rec-CRY2(535) (expressing GalAD-CRY2(535)). Under these conditions, wild-type CRY2 interacted with both CIB1 and wild-type CRY2 only when illuminated with blue light, but not in the dark. Confirming the high-throughput sequencing results, all tested enriched variants showed interaction with CIB1 in both light and dark (Fig. 1*G*, top), and no growth with the GalAD empty vector control lacking an insert (Fig. S1*A*). The K375R variant, as a non-enriched control, behaved similarly to wild-type CRY2 in interaction with CIB1, with growth only in light and not in the dark (Fig. 1*G*). Additional negative controls of GalBD empty vector and GalAD empty vector, or a GalBD empty vector with GalAD-CIB1, also showed no growth (Fig. S1*B*).

In contrast to the CIB1 interaction results, only four variants (D393S, D393A, M378R, and the positive constitutive control W374A) showed constitutive (dark) interaction with GalAD-CRY2(535) wild-type, while all tested variants showed interaction with CRY2 in blue light (Fig. 1*G*, bottom). To follow up on these results, we generated D393S, D393A, M378R, and W374A also in the GalAD vector, pGADT7rec-CRY2(535), allowing us to test mutant-mutant self-interactions (in contrast to testing interaction with wild-type CRY2) (Fig. 1*H*). D393S, D393A, M378R, and W374A all showed enhanced dark self-interaction, as measured using a quantitative GalUAS-lacZ reporter assay. The overall folds of AlphaFold3 models (34) for D393S, D393A, M378R, W374A and wild type are similar (Fig. S2), suggesting that these mutations do not cause extensive structural aberrations leading to nonspecific CRY2-CRY2 interactions. Taken together, our results suggest that the Region I variants D393S, D393A, and M378R (Fig. 1*F*) adopt conformations that mimic the photoactive state of CRY2. In contrast, for all other tested enriched variants, we detected constitutive interaction with CIB1 but not constitutive CRY-CRY interactions.

### Characterization of hyperactive CRY-CRY variants in mammalian cells

**Figure 2:**
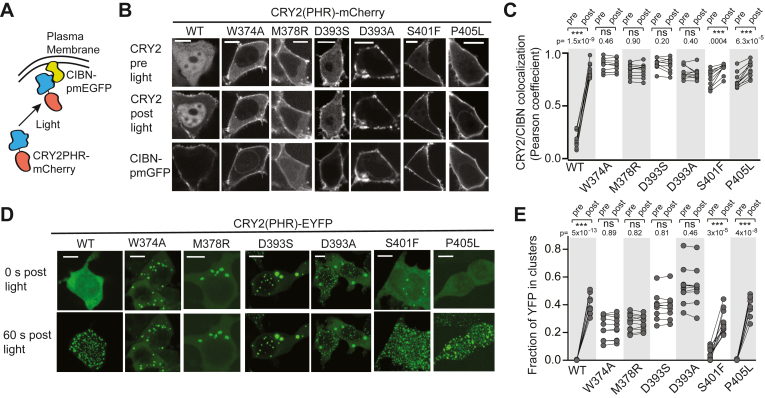
**Characterization of hyperactive variants in mammalian cells.***A*–*C*, membrane recruitment assay allowing visualization of interaction of CRY2 with plasma membrane (PM)-anchored CIBN in *dark* and *light*. *A*, schematic of experiment. *B*, representative images of HEK293T cells expressing CIBN-pmEGFP and indicated CRY2PHR-mCherry variants before and 60 s post *blue* light exposure. Localization of CIBN-pmEGFP is shown at *bottom*. Scale bars, 10 μm. *C*, quantification of recruitment as in *B*, showing the Pearson’s coefficient of co-localization of CIBN-pmEGFP at the membrane with indicated variants of CRY2PHR-mCherry, pre and post light. Wild-type CRY2PHR shows minimal co-localization with CIBN in dark, but strong co-localization at the PM with light; variants W374A, M378R, D393S, and D393A show pre-localization at the PM in dark and no additional light-induced recruitment. P405L and S401F show pre-recruitment in dark and additional recruitment with light. n = 10 cells quantified for each variant from two biological replicates. ns, not significant (*p* > 0.05); ∗∗∗, *p* < 0.001, two-tailed paired *t* test. *D*, EYFP clustering assay. Representative images of HEK293T cells expressing indicated CRY2PHR-EYFP variants before and 60 s after *blue* light exposure. Scale bars, 10 μm. *E*, quantification of clustering of indicated variants in CRY2PHR-EYFP before and 60 s after light exposure. 10 cells quantified from two biological replicates. ns, not significant (*p* > 0.05); ∗∗∗, *p* < 0.001, two-tailed paired *t* test.

To further confirm the D393S, D393A, and M378R constitutive phenotypes, we tested each mutation in mammalian cells using a plasma membrane recruitment assay and EYFP clustering assay. For comparison, we also characterized wild-type CRY2, the previously studied W374A mutant, and two mutants that showed constitutive CIB1 interaction, but not constitutive CRY-CRY interactions in yeast two-hybrid experiments: P405L and S401F. The membrane recruitment assay (Fig. 2*A*) monitors recruitment of CRY2PHR-mCherry to a plasma membrane localized CIBN (residues 1–170 of CIB1, CIBN-pmEGFP), allowing direct visualization of light/dark differences (4). In the dark, wild-type CRY2PHR-mCh shows minimal colocalization with CIBN at the plasma membrane, but becomes enriched at the plasma membrane with blue light (Figs. 2, *B* and *C*, S3*A*, and Video S1). M378R, D393A, D393S, and W374A variants all showed light-independent plasma membrane localization and no further increase in recruitment with blue light (Figs. 2, *B* and *C*, and S3*A*), consistent with a hyperactive state that is not responsive to light. In contrast, P405L and S401F showed light-independent membrane localization, but further recruitment to the plasma membrane in response to light. We hypothesize that further recruitment is due to enhanced CRY-CRY oligomerization that occurs with blue light, resulting in additional accumulation of CRY2 at the CIB-anchored site.

Constitutive activity of M378R, D393S, D393A, and W374A was further confirmed using an EYFP clustering assay. CRY2PHR-EYFP is diffuse in the dark but forms visible oligomeric puncta when illuminated with light, due to the valency increase from light-dependent tetramer formation and the weak dimer character of EYFP (35). We expected that mutations that induce constitutive tetramerization would result in puncta formation even without light treatment. Indeed, D393S, D393A, M378R, and W374A versions of CRY2PHR-EYFP showed pre-formed puncta in the dark, even without blue light exposure, and no further increases in puncta formation with light application (Figs. 2, *D* and *E*, S3*B*, and Video S2). In contrast, wild-type, P405L, and S401F versions of CRY2-EYFP showed robust puncta formation after exposure to blue light. While wild-type and P405L variants rarely showed any preformed puncta in the dark, some cells expressing S401F contained preformed EYFP puncta, which increased greatly upon light exposure. The preclustered S401F CRY2PHR-EYFP could represent misfolded or aggregated protein, or an increased propensity of this variant to self-associate in darkness (though at a level below that required to detect homomers in yeast two-hybrid experiments). Overall, our studies in mammalian cells show D393S, D393A, M378R, and W374A exhibiting constitutive behaviors and a lack of light-response, in clear contrast to the behavior of wild-type CRY2 in similar assays.

### *In vitro* characterization of the D393S variant in pCRY

**Figure 3:**
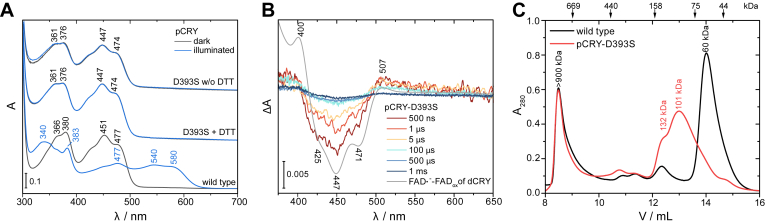
**Molecular characterization of pCRY-PHR-D393S *in vitro*.***A*, UV-vis absorbance spectra of D393S in the *dark* or illuminated with *blue light* for 30 s. In the presence and the absence of DTT, even excessive illumination of D393S does not result in the formation of a FAD neutral radical as observed for wild type. *B*, time-resolved UV-vis difference spectra of D393S. Excitation of D393S with a 10-ns laser pulse at 355 nm leads to a transient formation of FAD anion radical. Most of the radical decayed back to the oxidized FAD within 1 ms. The difference spectrum of FAD anion radical of *Drosophila* cryptochrome (dCRY) is shown for comparison (36). *C*, size-exclusion chromatography of D393S and wild type in the *dark*. D393S elutes mainly at 13 ml with an apparent molecular mass of 101 kDa close to a dimer, whereas wild type elutes as a monomer at 14 ml with an apparent molecular mass of 60 kDa. The excluded volume signal at 8.5 ml does not contain CRY protein. Elution volumes of standard proteins for calibration are indicated with *arrows* and with the respective molecular weight. From *left* to *right*: thyroglobulin (669 kDa), ferritin (440 kDa), aldolase (158 kDa), conalbumin (75 kDa), ovalbumin (44 kDa).

A unifying aspect in the activation of plant cryptochromes is the formation of the FAD neutral radical including a deprotonation of the conserved aspartic acid D393. Therefore, the mutation D393S directly interferes with a key step of the activation of plant cryptochromes. To study the photoreaction of the D393S variant, we introduced the mutation D393S into the PHR domain of the plant cryptochrome from *Chlamydomonas reinhardtii* (pCRY-D393S). pCRY is a close homolog of CRY2, with 48% sequence identity within the first 500 amino acids (PHR domain), and contains a conserved D393 residue. For *in vitro* characterization of the D393S mutant, switching from CRY2 to pCRY was necessary as studies on isolated CRY2 variants are challenging (30). UV-vis spectra of the mutant pCRY-D393S did not show any photoconversion of the oxidized FAD either in the presence or the absence of reducing agent DTT, in contrast to wild-type pCRY, which forms a stable FAD neutral radical (Fig. 3*A*). The transient photoproducts of pCRY-D393S were investigated with time-resolved UV-vis spectroscopy in the presence of ATP. Excitation of pCRY-D393S with a 10-ns laser pulse leads to a loss of oxidized FAD and the formation of anion radical (32, 36) from time points of 500 ns to 5 μs (Fig. 3*B*). After 500 μs, most of the photoproduct had decayed back to the oxidized FAD, whereas the wild-type pCRY shows formation of a long-lived neutral radical on these time scales (Fig. S4). Overall, spectroscopic characterization of pCRY-D393S showed that the chromophore only transiently reacts to light, of which the response decays too fast within 1 ms to be of any physiological consequence (16).

Based on the response of the FAD chromophore, D393S seemed to be inactive to light. However, another hallmark of cryptochrome activation is the formation of homomers by light. Accordingly, wild type and D393S were subjected to size exclusion chromatography in the dark in the presence of ATP (Fig. 3*C*). The main elution signal of wild type is observed at 14 ml, corresponding to an apparent molecular mass of 60 kDa according to calibration, which is supported by SDS-PAGE analysis (Fig. S5). Comparison to the theoretical mass of 59 kDa indicates that pCRY is monomeric in the dark. The main elution signal of D393S is shifted by 13 ml to a higher apparent mass of 101 kDa, close to a dimer. These enhanced CRY-CRY interactions of D393S in the dark support a hyperactive state of the variant. The elution signal is broadened with a shoulder at 132 kDa and contains only pCRY according to SDS-PAGE analysis (Fig. S5). Accordingly, the signal hints toward dynamic CRY-CRY interactions of D393S in the dark, in agreement with results from two-hybrid assays (Fig. 1*G*). Dimer formation was also observed by size exclusion chromatography in a previous study on constitutively active CRY2 W374A (29). Taken together, *in vitro* characterization of D393S indicates a decoupling of FAD and protein moiety, because the FAD fails to form a stable photoproduct, whereas the protein adopts homomers resembling an activated state.

### Characterization of region II variants in the CRY2PHR background

**Figure 4:**
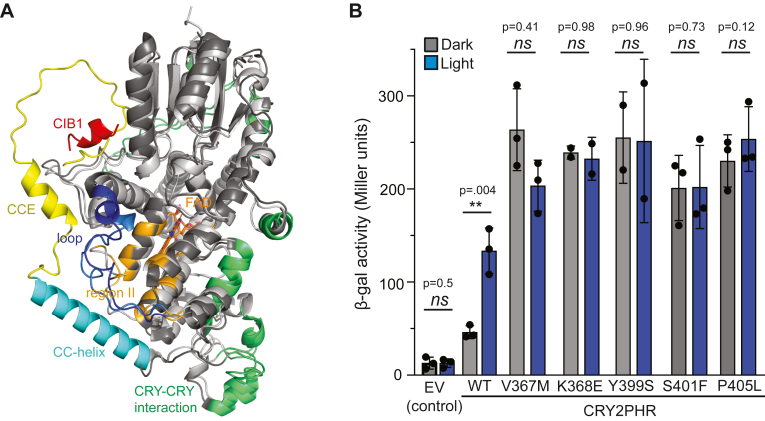
**Model of CRY2(1–535) and characterization of role of CCE tail in hyperactivity of region II variants.***A*, the overlay of an AlphaFold3 model of CRY2 (1–535, *light gray*) and the cryo-EM structure of constitutively active CRY2-W374A with CIB1 bound (PDB: 6X0Y, dark*gray*) suggests that the CCE (*yellow*) binds to the CIB1 binding site, potentially competing with CIB1 (*red*). Region II (*orange*) identified in this work is adjacent to the CC-helix and separated from the CIB1 binding site only by a loop region (*blue*). Besides CIB1 binding, activation of CRY2 leads to tetramerization *via* CRY-CRY interactions (*green*). Five best AlphaFold3 models and pLDDT values (33) are shown in Figure S7*A*. *B*, yeast two-hybrid experiments testing the effect of CCE tail loss on constitutive interaction with CIB1. Graph shows beta-galactosidase reporter activity (Miller Units) resulting from CRY2PHR interaction with CIB1 in yeast strain AH109 x Y187, using wild-type CRY2 (WT) or indicated hyperactive mutants. In all cases, tested hyperactive region II variants show constitutive interaction with CIB1 beyond the levels observed with CRY2PHR, indicating the mutations do not merely mimic the CRY2PHR (residues 1–498) truncated state. Yeast were exposed to *light* or *dark* for 2 h. Samples labeled EV (empty vector) express the Gal4BD vector with no insert, tested for interaction with GalAD-CIB1. Graph shows mean and error (s.d) with individual biological replicates, ∗∗, *p* = 0.004; ns, not significant (*p* > 0.05), two-tailed unpaired *t* test.

Studies of *Drosophila* cryptochrome (dCRY) have suggested the CCE tail can dock onto the PHR domain in the dark, preventing binding to effectors (37, 38), undocking in light to a more open conformation (38, 39, 40). Similar docking/undocking of the CCE on the PHR domain has been suggested for plant cryptochromes (13, 16, 19). Supporting this idea, we previously found that truncations of the CCE of CRY2 resulted in enhanced dark-dependent interaction with CIB1, suggesting the CCE may interfere with CIB1 interaction in the dark (25). In the CRY2 structure, Region II is adjacent to helix α21 (CC-helix), and we hypothesized that mutations to this region could affect docking of the short CCE extending from α21, leading in turn to enhanced CIB1 binding (Fig. 4*A*). To test this possibility, we transferred a subset of the identified CRY2 Region II variants into the BD-CRY2PHR construct lacking the CCE (residues 1–498 of CRY2) and tested interaction with AD-CIB1 (Fig. 4*B*). In the dark, wild-type CRY2PHR indeed showed enhanced baseline (dark) interaction with CIB1 as compared with CRY2(1–535), but also a strong light-dependent response, where after 2 h light illumination, interaction was increased 4-fold over dark levels. In comparison, Region II mutations (V367M, K368E, Y399S, S401F, P405L) placed in the CRY2PHR background showed 4- to 7-fold higher basal interaction with CIB1 in the dark, and no light-dependent changes in interaction (Fig. 4*B*). Our results showing enhanced CIB1 binding in the dark beyond that observed with CRY2PHR indicate that the structural changes induced in Region II do not merely mimic the CRY2PHR truncation but act distinctly to affect the CIB1 interface.

## Discussion

In this study, we undertook a comprehensive screen for CRY2 variants that undergo enhanced interaction with CIB1 in the dark, identified using a yeast two-hybrid and deep mutational scanning approach. To validate the high-throughput screen results, we selected 10 enriched variants for further analysis, independently confirming the initial results showing enhanced interaction with CIB1 in the dark. Strikingly, only three of these variants also showed constitutive CRY-CRY self-interaction. Thus, our study allowed separation of hyperactive mutants into two classes: one class showing constitutive CIB1 interaction only, and the other class showing constitutive CRY-CRY as well as CIB1 interaction. In the latter category, we characterized three mutations located at D393 and M378 near the FAD isoalloxazine ring (D393S, D393A, and M378R) using yeast and mammalian cell assays. All three variants, as well as a W374A control, showed constitutive CRY-CIB1 and CRY-CRY activity in the dark and no observed light-dependent changes in independent assays, consistent with the adoption of the photoactivated state in the dark.

Our initial screen identified other highly enriched variants at D393 and M378, including M378T, M378K, M378S, and D393G. While we did not independently validate all of these, we suspect that each will show a similar constitutive phenotype as D393S, D393A, and M378R. One other highly enriched variant deserves mention: M378∗, containing a substitution to an amber stop codon at M378, which scored as significantly enriched in 3/3 screens. Previous studies have shown that stop codons can be suppressed in yeast through incorporation of a near-cognate tRNA, with the amber UAG codon replaced by tRNA encoding K, Q, and Y (41). Given the high enrichment score of the M378K variant, this suggests the potential that the yeast engage in non-optimal tRNA recognition at M378, substituting the near-cognate K, resulting in stop codon suppression and resulting in enhanced growth similar to the M378K phenotype.

**Figure 5:**
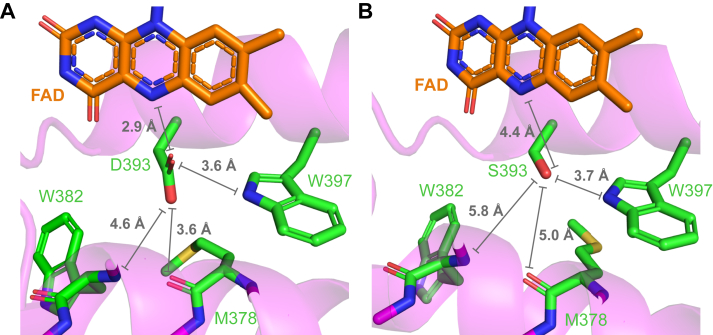
**Structure of the active site of the wild type and model of the D393S mutant.***A*, distances of D393 to hydrogen bonding partners and polar neighbors within the active site of CRY2 are indicated in the *crystal* structure of the PHR (PDB entry 6K8I). *B*, distances of S393 to polar neighbors are much larger than for D393 suggesting a loss of hydrogen bonds and resulting decoupling of FAD and protein moiety. The distances were determined from an AlphaFold3 model (33) of the D393S mutant. Five best AlphaFold3 models and pLDDT values are shown in Figure S7, *B* and *C*.

We followed up our initial findings on CRY2 D393 with *in vitro* studies in pCRY, a homologous cryptochrome, using time-resolved UV–vis spectroscopy and size exclusion chromatography. We found that constitutive activation by D393S can be transferred to pCRY, emphasizing the central role of D393 in light activation and supporting a unifying activation mechanism in plant cryptochromes. The D393S mutant forms homomers in the dark with oxidized FAD bound. This finding supports an uncoupling of the activated protein moiety from the oxidation state of FAD in the dark. Upon illumination, the FAD neutral radical as a signaling state does not form because the aspartic acid responsible for the protonation of the FAD anion radical is mutated to a serine (15). At this point, it can be speculated about the structural origin of D393S constitutive activation. The aspartic acid is close to two hydrogen bonding partners, W397 and the backbone of M378, in the dark state structure of CRY2PHR (Fig. 5*A*). The introduced serine at D393 is sterically less demanding than the aspartic acid, and the distances, especially to the backbone of M378, are considerably increased (Fig. 5*B*). This increase causes a disruption of hydrogen bonds within the active site. The constitutive activity might therefore result from destabilization of the CRY2 dark structure. This model is supported by constitutively active mutants D393A (Fig. 1*G*) and D393G (31), which cannot form hydrogen bonds.

Several hyperactive variants have been initially identified in plants showing phenotypes of early flowering during short days, including CRY2 D393G, S401F, V367M, and CRY1 L407F and G380R (equivalent to CRY2 I404F and G377R) (30, 31, 33, 42). With CRY2 I404F (30), the FAD cofactor was found to undergo light-independent reduction in the presence of reducing agent, which was not observed with wild-type CRY2 and in clear contrast to D393S in this study. These differences suggest a FAD-based hyperactivity of I404F, whereas in D393S the coupling of FAD and protein moiety might be lost so that the formation of the active CRY2 structure is preferred over the inactive structure. Such a scenario would imply that oxidized FAD acts as a repressor for formation of the active CRY2 structure in the wild type in the dark and repression is released by photoreduction.

All of the variants showing enriched dark CRY2-CIB1 interaction in the initial screen also showed constitutive CRY2-CIB1 interaction in independent two-hybrid experiments. However, only a subset of these also showed constitutive CRY-CRY interaction, suggesting two classes of constitutive mutants. Based on our high-throughput data (Fig. 1*D*), substitution of the amino acids K368, I394, Q398, and Y399 results in constitutive interaction with CIB1 independent of the introduced amino acids, suggesting a crucial role of these residues in maintaining the dark state structure of CRY2. These and other amino acids that are important for preventing CRY-CIB1 interactions in the dark are located around FAD and the C-terminal helix (CC-helix; helix α21 of CRY2) of the PHR domain (Fig. 1*F*). However, the documented CIB1 interaction site of CRY2 is at a distance of 20 to 30 Å from the FAD and CC-helix (28). Light-induced dissociation of the CCE from the PHR was proposed for plant cryptochromes (13, 16, 19), and therefore, the light-induced signal might progress from the CC-helix to the CCE, releasing the CCE from the PHR. Hence, the CCE might block the CIB binding site in the dark and expose the site upon illumination. This model agrees with enhanced CRY-CIB1 interactions in CCE-truncated CRY2 constructs, in which CIB1 binding can be further enhanced by light. Accordingly, the affinity of CIB1 to CRY depends on the accessibility of the CIB1 binding site and additional multivalent interactions promoted by light-induced structural changes in the PHR. The signal within the PHR might be transmitted from Region II to the CIB1 binding site, as these sites are separated only by a loop region (Fig. 4*A*).

Overall, our study demonstrates the utility of applying deep mutational scanning methods to characterize constitutively active photoreceptor states and identifies two classes of hyperactive CRY2 variants: those that bind CIB1 in the dark but remain light-sensitive for CRY homomeric complexes, and those that show light-independent binding to both CRY and CIB1. Accordingly, after initial light activation, signal progression might split into two pathways for CRY-CIB1 and CRY-CRY interactions. We note that this initial study was focused on the identification of CRY-CIB1 interactions, however, a targeted screen for constitutive CRY-CRY interactions could also be useful in revealing the pathway of signal progression responsible for CRY-CRY interaction. Our screen was restricted to residues 321 to 412 of CRY2 near the FAD; therefore, future screening could extend this approach to target additional regions. Given the demonstrated differences in activation pathways for CRY-CIB1 and CRY-CRY interactions for the variants identified, their characterization in plants may provide additional valuable insight.

## Experimental procedures

### Plasmids and yeast strains

Yeast strains used for two-hybrid assays were MaV203 (MATα; leu2-3112; trp1-901; his3Δ200; ade2-101; cyh2R; can1R; gal4Δ; gal80Δ; GAL1::lacZ; HIS3UASGAL1::HIS3@LYS2; SPAL10::URA3), AH109 (*MATa, trp1-901, leu2-3, 112, ura3-52, his3-200, gal4*Δ, *gal80*Δ, *LYS2:: GAL1UAS-GAL1TATA-HIS3, GAL2UAS-GAL2TATA-ADE2, URA3:: MEL1UAS-MEL1TATA-lacZ*), and Y187 (*MAT*α*, ura3*-*52*, *his3*-*200, ade2*-*101*, *trp1*-*901, leu2*-*3*, *112*, *gal4*Δ, *met–*, *gal80*Δ, *URA3:: GAL1*UAS*-GAL1*TATA*-lacZ*). Plasmids used in two-hybrid screening were pGADT7rec-CIB1, pDBTrp-CRY2PHR, pDBTrp-CRY2(535), and pGADT7rec-CRY2(535) (4, 25). Empty vector controls used were pGADT7rec and pDBTrp without inserts (meaning no protein was fused to Gal4AD or Gal4BD, respectively). CRY2 mutations selected for independent verification of the sequencing results and further analysis were separately created by PCR using mutagenic oligonucleotides (provided in Fig. S8). For each mutation, we used PCR to generate two fragments that overlapped at the mutagenic region by 25 to 30 bp. Yeast were transformed with the two fragments and a gapped version of pDBTrp-CRY2(535) to generate the mutations by yeast homologous recombination. The pDBTrp-CRY2PHR mutants were generated from pDBTrp-CRY2(535) by PCR using an oligonucleotide that truncated CRY2 at residue 498 (oligos 5′-ACAAAGGTCA AAGACAGTTG ACTGTATCGC CGCTCGAGGC CACCATGAAG ATGGACAAAA AGACTAT-3′ and 5′-TCGCCCGGAA TTAGCTTGGC TGCAGGTCGA CCCGGCTGCT GCTCCGATCA TGATCT-3′), followed by yeast homologous recombination into pDBTrp. For plasma membrane recruitment studies in mammalian cells, variants were amplified from CRY2(535) by PCR (oligos 5′-CTTGGC TCGAGG CCACCA TGAAGA TGGACA AAAAGA CTATAG TTTGG-3′ and 5′-TTCGCC CGGAAT TAGCTT GGCTGC AGGTCG ACCCGG GCTGCT GCTCCG ATCATG ATCT-3′) followed by ligation at XmaI and XhoI sites in CRY2PHR-mCherry. CIBN-pmEGFP was described previously (4). The EYFP clustering assay used CRY2PHR-EYFP, generated by ligating the CRY2PHR insert (cut from CRY2PHR-mCherry with XhoI and XmaI restriction enzymes) into EYFP-N1 vector (Clontech) at XhoI and XmaI sites. Individual CRY2 mutant versions were then cloned into the EYFP vector to replace wild-type CRY2PHR.

### Yeast deep mutational scanning and selection

To generate a mutagenic library for deep mutational scanning, error-prone PCR was performed on CRY2 between amino acids 319 and 412 (average 1.9 mutations for each DNA fragment) using a MnCl_2_/unbalanced nucleotide protocol (25). We performed PCR reactions using Taq DNA polymerase in the presence of 2.5 mM MgCl_2_, 0.25 mM MnCl_2_, 1 μM each primer, 1x Taq buffer, 1 mM dTTP and dCTP, and 0.2 mM dGTP and dATP. To insert the mutagenic library, we first made a non-functional GalBD-CRY2 fusion in the vector pDBTrp-CRY2(1–535) containing a mCherry stuffer inserted between residues 329 and 409 of CRY2 (B1732). The library of variant inserts was cotransformed with the stuffer vector B1732, digested with Nde I and Xma I, into MaV203 yeast already containing pGADT7rec-CIB1 and plated onto SC –Trp/–Leu to select for both bait (pDBTrp) and prey (pGADT7rec) plasmids. The initial titer, representing unique recombination events, was 500,000 colonies. For each selection, approximately five million yeast were plated on SC –Trp/–Leu/–Ura in the dark to screen for constitutive CRY2-CIB1 interaction. Screen one was plated for 48 h. For Screen 3 and Screen 4 (96h screens), yeast were plated on SC –Trp/–Leu/–Ura in dark for 48 h, then yeast were pooled and replated in the dark on SC –Trp/–Leu/–Ura for an additional 48 h then pooled and sequenced.

### Sequencing and data analysis

For sequencing, yeast from each selection were pooled, and PCR was performed using a barcoded forward primer (XXX-TCTTCGGTTTTTTCCC) and a reverse primer (TCCAAGCGATCAAGC), which amplified a 300-base region of the CRY2 gene targeted during mutagenesis. The "XXX" represents a three-nucleotide barcode used to enable multiplexing during sequencing. To preserve library diversity, PCR reactions were conducted in eight separate tubes for each selection step and subsequently combined after the reaction. The resulting PCR products were purified using agarose gel extraction and then sequenced at the University of Colorado Anschutz Genomics Core facility. The amplicon purity, quantity, and size distribution was determined with Qubit (Invitrogen) and TapeStation 4200 (Agilent) analysis prior to DNA-seq library preparation. The Ovation Ultralow System V2 kit (Tecan) was used with an input of 10 ng of the amplicon to generate DNA-Seq libraries. Paired-end sequencing reads were generated on NovaSeq X (Illumina) sequencer at a target depth of 100 million paired-end reads per sample. Raw sequencing reads were de-multiplexed using bcl2fastq. Sequences consisted of 149 to 151 bp forward and reverse reads, with a small (7 bp) gap in between corresponding to S363-F365. Thus, this region was not included in our analysis. After sequencing, low-confidence sequences were filtered out by applying a minimum PHRED score threshold of 10.

Sequencing data were analyzed using Enrich2 (43). To identify high-confidence enriched variants, we applied the Enrich2 computational tool to each independent screen. For each screen, the data was normalized to wild-type counts using Enrich2. The Enrich2 software calculates a variant score (S_var_) based on the natural log of variant ratios before and after selection, in which samples that are greater than 0 are enriched and those less than 0 depleted, as compared to the original library pool. The software also calculates a standard error (SE) for each score. For each variant in each screen, we calculated the Z value as absolute value of (S_var_-S_wt_)/(√((SE_var_)^2^ +(SE_wt_)^2^)) and applied filtering to exclude all variant scores with Z < 3.29 (99.7% confidence). We designated the 50 variants with the highest S_var_ for each replicate screen as “highly enriched.”

### Live cell imaging

HEK293T cells were maintained in Dulbecco′s Modified Eagle′s Medium (DMEM) with 10% FBS at 37 °C with 5% CO_2_. Cells were plated on 35-mm glass-bottom imaging dishes and transfected using Lipofectamine 2000 (Life Technologies) according to the manufacturer's protocol. Cells were incubated in dark (wrapped with foil) and manipulations were carried out under dim light or using a red safelight. Before imaging (24 h after transfection), cells were moved to Hepes-buffered saline (HBS) with 1 mM CaCl_2_. Live cell imaging was performed at 33.5 °C using an Andor Dragonfly 301 spinning disc imaging system, with an Olympus IX73 base and four-line ILE laser merge module and controller. Images were acquired using a 60x UplanSApo 1.35 NA oil objective and collected on a 1024 × 1024 pixel Andor iXon EM-CCD camera. Data were acquired using Fusion 2.3 or iQ3.6 software (Oxford Instruments).

### Image analysis

All images were analyzed using ImageJ software. For membrane recruitment studies, individual Z-planes were used for analysis, while clustering experiments used a max projection image consisting of compiled Z-stacks (10 stacks, 1 μm each) processed using the ImageJ Z project function. For all quantifications, mean background fluorescence was subtracted. To quantify CRY2 membrane recruitment, we calculated the Pearson’s coefficient for colocalization between CRY2PHR-mCh and CIBN-pmEGFP within a rectangular region of the cell that encompassed both the membrane and a portion of the cytosol, using the ImageJ plugin JACoP (44) with the “Costes” automatic threshold. To quantify the percentage of clustered CRY2(535)-EYFP, for each cell, we calculated the total fluorescence F_t_ = area∗mean background subtracted fluorescence. The total fluorescence within clusters was then specified using automatic thresholding in ImageJ (Otsu method) to specify clustered regions, then calculating F_c_ = clustered area∗mean background subtracted fluorescence in clusters. The ratio F_c_/F_t_ was then reported for Figure 2*E*. Plots were generated using GraphPad Prism 10.

### Yeast-two hybrid (growth and ONPG assays)

For mating screens, variants in pDBTrp-CRY2(535) were transformed into strain AH109, with Gal4AD fusions (CIB1 or CRY2(535) variants in pGADT7rec) transformed into strain Y187 (Clontech). AH109 and Y187 strains were mated and selected on SD–Trp/–Leu plates to generate diploid AH109 × Y187 strains containing both AD and BD plasmids. To assess interaction, diploid strains were plated on selective media (SD –Trp/–Leu/–His + 3mM 3-AT) and incubated in dark or in blue light (1 s pulse every 3 min, 465 nm, 4.7 mW/cm^2^) for 3 days. To quantify beta-galactosidase reporter activity, yeast strains were grown overnight in dark in SD –Trp/–Leu media, then diluted to 0.2 OD_600_ the next morning. Following an initial 3 h growth period in the dark, cultures were either kept in the dark or exposed to a 465 nm LED light source for 2 to 3 h (1s pulse every 3 min) as indicated in figures. After light treatment, cultures were harvested and lysed with Y-PER reagent (Thermo Scientific), then assayed for β-galactosidase activity (Clontech Laboratories, protocol #PT3024–1) using ONPG (Sigma-Aldrich) as a substrate.

### Protein expression and purification

pCRY-PHR D393S (amino acids 1–504) was expressed with an N-terminal His_6_ tag and a C-terminal StrepII tag in *E. coli* BL21 (DE3) using a pET-28a(+) vector. Protein expression and cell harvesting was done as described previously (45). Cells were lysed *via* French press in 50 mM phosphate buffer pH 7.8, 100 mM NaCl, 20% glycerol containing one tablet protease inhibitor (Complete EDTA-free, Roche Applied Science) and DNase I. After centrifugation of the lysate (100,000 rcf, 1 h, 4 °C), the supernatant was loaded on a Strep-Tactin-Sepharose column (IBA Lifesciences) and washed with phosphate buffer at 4 °C. pCRY-PHR D393S was eluted with phosphate buffer containing 2.5 mM d-desthiobiotin. The protein was washed with 50 mM phosphate buffer, 100 mM NaCl, 1% glycerol, at pH 7.8, concentrated and stored at −80 °C for further experiments.

### Static UV-vis spectroscopy

UV-vis spectra of pCRY-D393S and wild-type pCRY were recorded with a Shimadzu UV-2450 spectrometer at 10 °C. All protein samples were buffer exchanged to a 50 mM phosphate buffer with 100 mM NaCl, 3 mM ATP and 1% glycerol at pH 7.8. For the measurements in the presence of external reductant, DTT was added to the buffer to a concentration of 2 mM. Illumination experiments were performed using a quartz cuvette (Suprasil, Hellma) with a 10 mm optical path length. The samples were illuminated with a blue LED (454 nm, 35 mW cm^−2^ at the sample, Philips Lumileds) for 30 s.

### Time-resolved UV–vis spectroscopy

For time-resolved UV-vis difference spectroscopy, a setup with an intensified CCD camera (ICCD, DH734I-18U-03, Andor iStar) cooled down to −30 °C, a spectrograph (Model 1235 Digital tripel grating spectrograph, EG&G Princeton Applied Research), a Xe-lamp (Oriel) as probe light and a pulsed Nd:YAG laser (355 nm, 6 mJ, 5 ns pulse width, 10 Hz, Quantel Ultra) for excitation was used. pCRY-D393S at a concentration of 30 μM in 50 mM phosphate buffer, pH 7.8, 100 mM NaCl, 1% glycerol and 3 mM ATP was placed in a fluorescence cuvette (10 × 10 mm, Hellma) and cooled to 13 °C. Two shutters controlled perpendicular excitation and probing of the sample. To minimize multiple excitations and ensure homogenous illumination, the sample was stirred with a magnetic stirrer between each measurement and a mirror was placed behind the sample reflecting the excitation light. Alternating intensity spectra of illuminated pCRY-D393S or without excitation were recorded every 1.6 s with gating times of 300 ns, 500 ns, 1 μs, 10 μs, 100 μs, and 100 μs for spectra at 500 ns, 1 μs, 5 μs, 100 μs, 500 μs, and 1 ms, respectively. For each measurement, a dark spectrum without excitation and probe light was recorded. The difference spectra resulted from an average of 32 individual measurements.

### Size exclusion chromatography

Size exclusion chromatography was performed *via* an NGC chromatography system (Bio-Rad) using a Superdex 200 Increase 10/300 Gl column (Cytiva). All steps were carried out under light exclusion at 4 °C, using a 50 mM Tris buffer at pH 7.8, with 100 mM NaCl, 1 mM ATP and 1 mM MgCl_2_. Protein samples of pCRY-D393S and pCRY-wt were centrifuged at 15,000*g* for 3 min and adjusted to an OD_450_ = 1. For each protein, 100 μl of sample was loaded onto the column previously equilibrated with two column volumes (CV) of buffer. The proteins were eluted at a flow rate of 0.3 ml·min^−1^ and the elution profiles were recorded at 280 nm. The apparent molecular masses were calculated from the elution volumes using a Gel Filtration HMW Calibration Kit (Cytiva).

### AlphaFold3 models

Structural models of CRY2 and D393S were generated using the AlphaFold3 server (https://golgi.sandbox.google.com/) (33). The protein sequence of CRY2 was taken from the UniProt server (https://www.uniprot.org/uniprotkb/Q96524/). For the D393S structure, aspartic acid 393 was substituted by a serine in the sequence. Structures were calculated with stoichiometric amounts of FAD and ATP as cofactors. Five models were generated and analyzed for structural integrity and for the predicted local distance difference test (pLDDT). Models are available in ModelArchive (https://modelarchive.org) DOI 10.5452/ma-l5i.e.9 and 10.5452/ma-gmb43 (46).

## Data availability

The data described in the manuscript is contained within the manuscript and Supporting Information. Raw fastq data for each screen is archived at Open Science Framework (https://osf.io/3axfg/) DOI 10.17605/OSF.IO/3AXFG.

## Supporting information

This manuscript contains supporting information, including Figures S1–S8, Supplementary Table Legends, and Supplementary Video Legends, Table S1, and Videos S1 and S2 (15, 34).

## Conflict of interest

The authors declare that they have no conflicts of interest with the contents of this article.
