# Supplementary material for: Constitutively active Arabidopsis cryptochrome two alleles identified using yeast selection and deep mutational scanning
Source: J Biol Chem. 2025 May 21;301(6):110265. doi: 10.1016/j.jbc.2025.110265 (PMC12212125; doi:10.1016/j.jbc.2025.110265)
Supplement: Supplementary Table S1 Legend [file mmc1.pdf]

Supplementary Table S1. Summary of variant enrichment scores and standard errors calculated using Enrich2 software. The table indicates variant scores, SE, and calculated Z values for each of three independent screens (Screen 1, Screen 3, and Screen 4). Note that Screen 2 was not included due to poor sequencing quality control. For each screen with a Z value of greater than 3.29, the top 50 enriched variants are highlighted in purple. Top50_Count for each variant indicates the total number of times (out of 3) that variant was enriched in top 50. Variants scoring in the top 50 enriched in at least 2 out of 3 screens are highlighted in green in the first column. Highlighted in light green are variants that showed enrichment in all three screens (but did not score in top 50 in 2), with those with Z-scores lower than 3.29 filtered out. AveScore is the average score from all three screens, and includes data from all screens including those with high error (with Z-score <3.29).
