# Supplementary material for: Constitutively active Arabidopsis cryptochrome two alleles identified using yeast selection and deep mutational scanning
Source: J Biol Chem. 2025 May 21;301(6):110265. doi: 10.1016/j.jbc.2025.110265 (PMC12212125; doi:10.1016/j.jbc.2025.110265)
Supplement: Supporting information [file mmc2.pdf]

Supporting Information

Constitutively active *Arabidopsis* cryptochrome 2 alleles identified using yeast selection and deep mutational scanning

[1]Amir Taslimi^, [2,3]Axel Jeibmann^, [2,3]Lukas Goett-Zink, [2,3]Tilman Kottke*, and [1]Chandra L. Tucker*

[1]Department of Pharmacology, University of Colorado School of Medicine, Aurora, CO 80045

[2]Biophysical Chemistry and Diagnostics, Medical School OWL, Bielefeld University, 33615 Bielefeld, Germany

[3]Biophysical Chemistry and Diagnostics, Department of Chemistry, Bielefeld University, 33615 Bielefeld, Germany

Contents:

Supplementary Figures S1-S8

Supplementary Video Legends

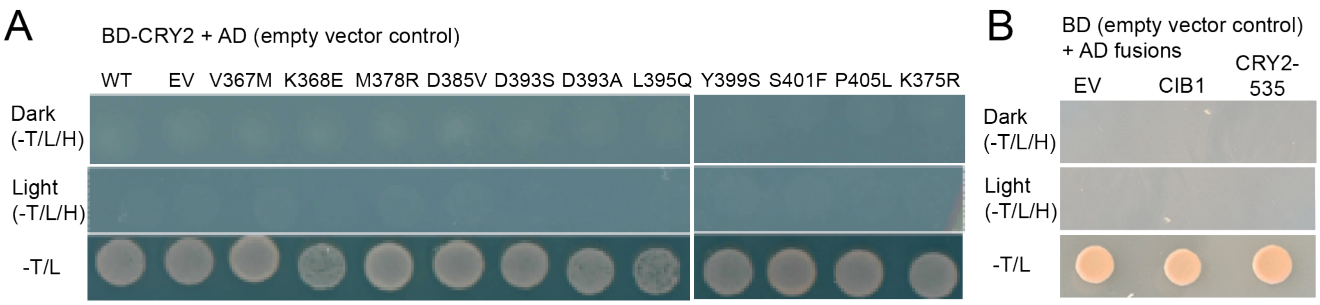

Figure S1. **Yeast two-hybrid controls showing CRY2 variants do not self-activate or interact with the activation domain (AD) or binding domain (BD) alone. (A)** Shown are AH109 yeast expressing each CRY2 bait construct with different mutations (pDBTrp-CRY2(535)) mated to Y187 yeast expressing a pGBKT7rec empty vector (with no protein fused to Gal4AD). The bottom row shows growth of diploid yeast containing both bait (GalBD fusion) and prey (GalAD fusion) on non-selective plates. The top and middle rows show growth on -Trp/-Leu/-His + 3mM 3AT plates exposed to light or dark, selecting for His3 reporter activation (indicative of interaction). **(B)** Additional two-hybrid controls prepared as in (A) showing lack of growth on selective (-Trp/-Leu/-His + 3mM 3AT) plates for samples including: GalBD-CRY2 + AD alone (empty vector pGADT7rec); GalBD alone (empty vector pDBTrp) + GalAD-CIB1; pDBTrp (empty vector only) + pGADT7rec (empty vector only). EV indicates empty vector; WT indicates wild-type.

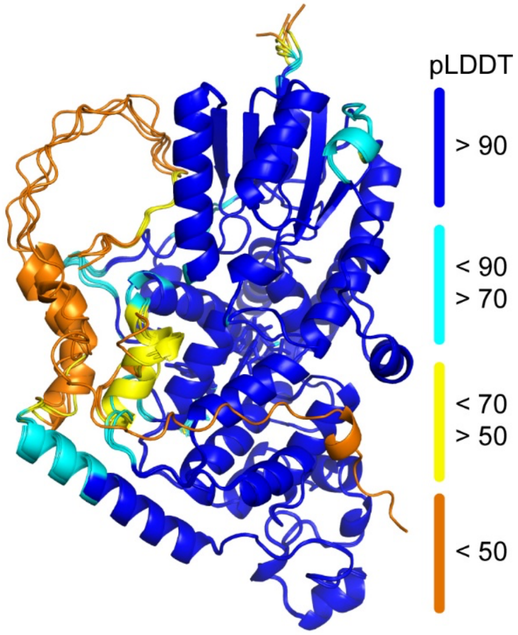

Figure S2. Structural models of wild-type CRY2(1-535) and the mutants D393S, D393A, M378R, and W374A generated by AlphaFold3. The prediction by AlphaFold3 does not indicate any larger structural alteration in the CRY2 mutants as compared to the wild type. The prediction confidence of the backbones for the models is indicated by the predicted local distance difference test (pLDDT) (34), a score that scales from 0-100, with scores >90 indicative of the highest confidence predictions.

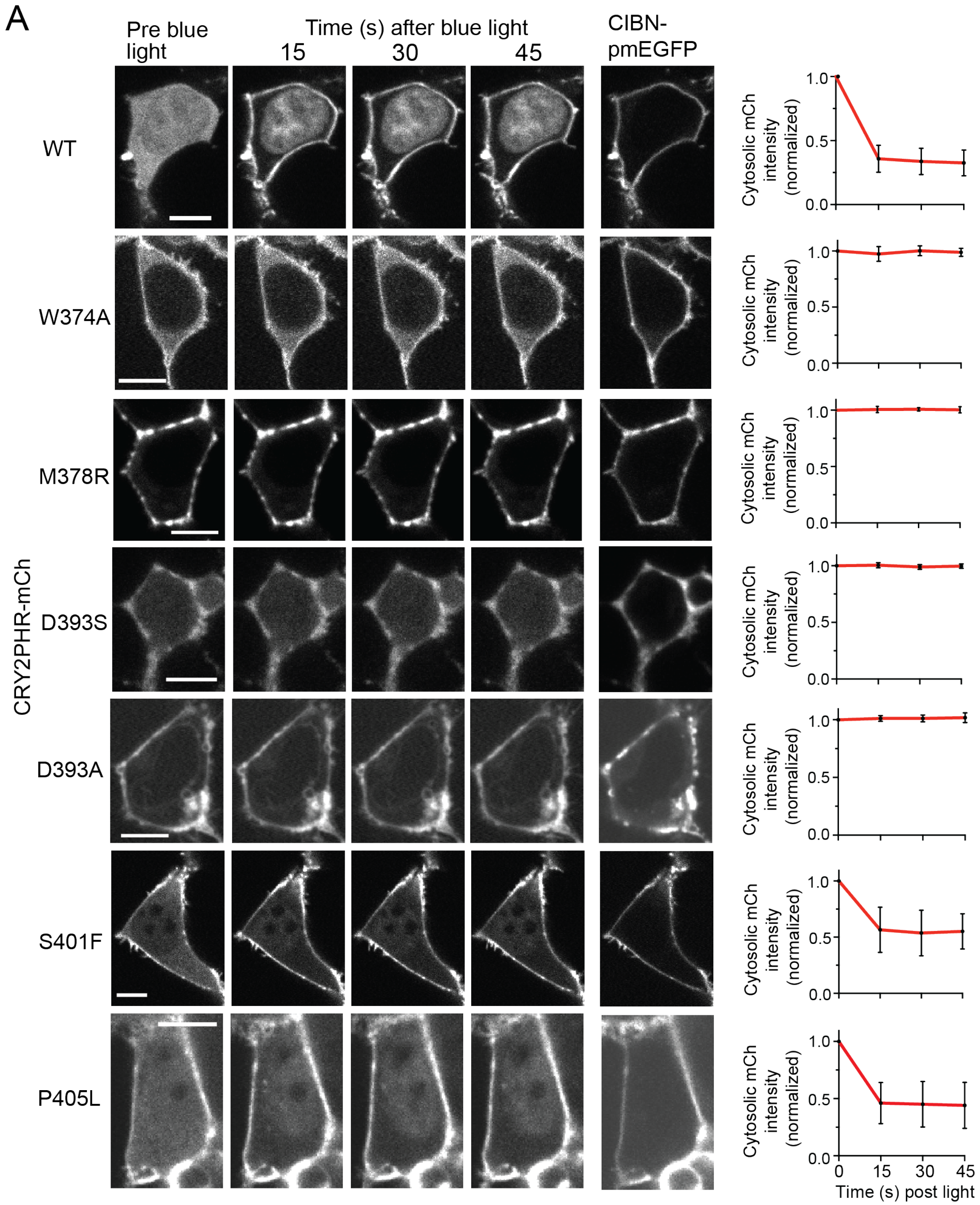

Figure S3. Time-lapse images and quantification of hyperactive CRY2PHR variants in mammalian cells. (A) Time-lapse images of representative HEK293T cells expressing CIBN-pmEGFP and indicated CRY2PHR-mCherry variants tested for light-dependent changes in membrane localization, similar to as described for Figure 2B. Note that for the cells shown in the D393A and S401F panels, the CRY2 pre blue light and CIBN-pmEGFP images are derived from the same source images as the CRY2 pre light and CIBN-EGFP images in Figure 2B. Cells were initially exposed to blue light at t=0, then imaged and light-stimulated every 15s. The column labeled CIBN-pmEGFP shows the localization of CIBN at the plasma membrane. Scale bars, 10 µm. Graphs at right show quantification of change in cytosolic CRY2PHR-mCherry signal in cells pictured at left. Values are normalized to the initial (time 0) value. Data represents mean and error (s.d.), n=10 cells from two biological replicates.

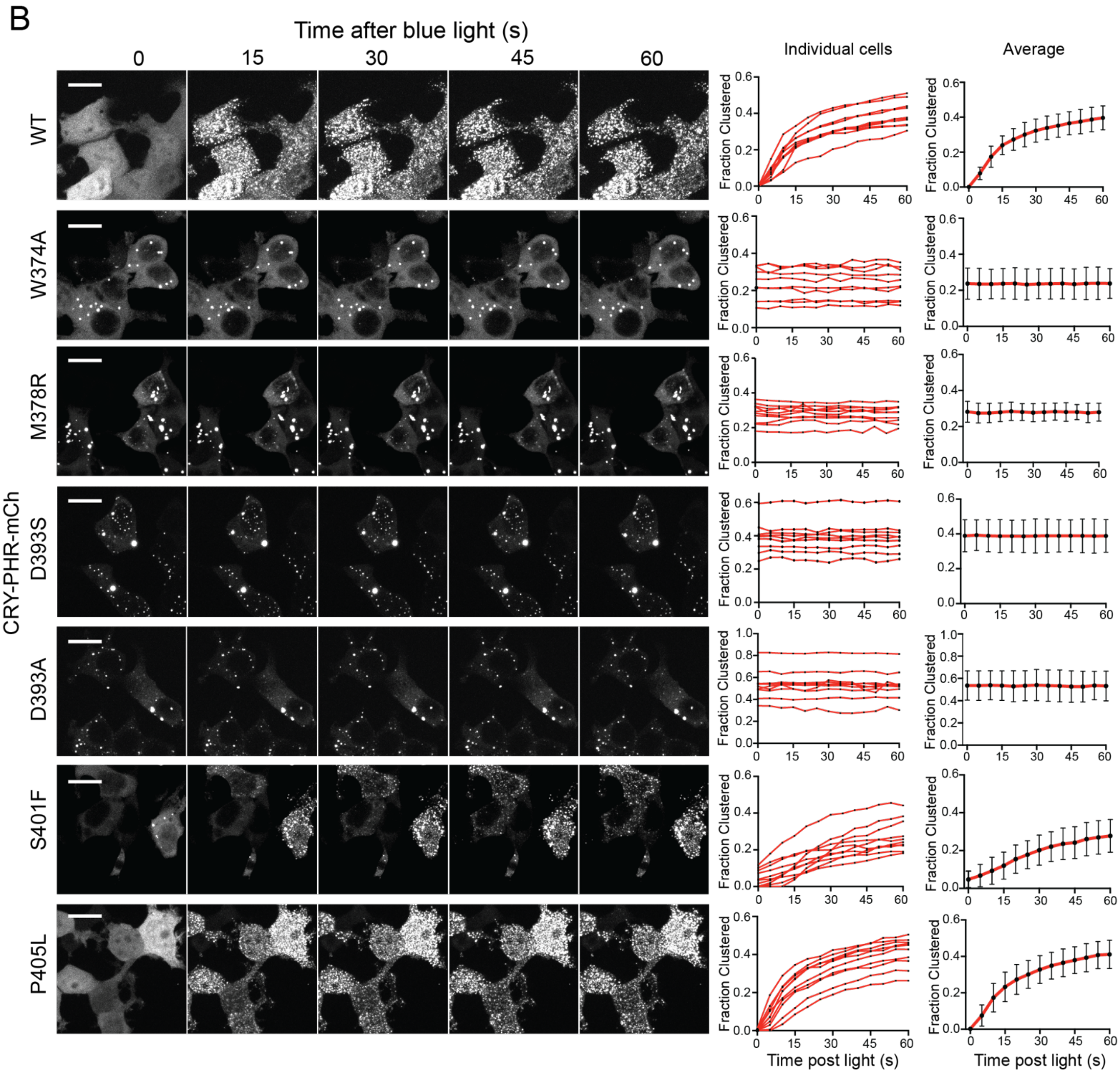

Figure S3 (continued). (B) Representative images (left) and quantification (right) of HEK293T cells expressing indicated CRY2PHR-EYFP variants before and up to 60 s after blue light exposure. Scale bars, 20 µm. Cells were initially exposed to 488 nm blue light at t=0 (with "0 s after blue light" representing the initial imaging capture that also serves to initiate light treatment), then imaged and light-stimulated every 5s for the duration of the timecourse. Graphs show quantification of percent of clustered CRY2PHR-EYFP over time after light exposure. Individual cell cluster trajectories are shown at left, with graph at right showing mean and error (s.d., n=10 cells). Data is from two biological replicates. Experiments were independently performed 3-4 times with similar results.

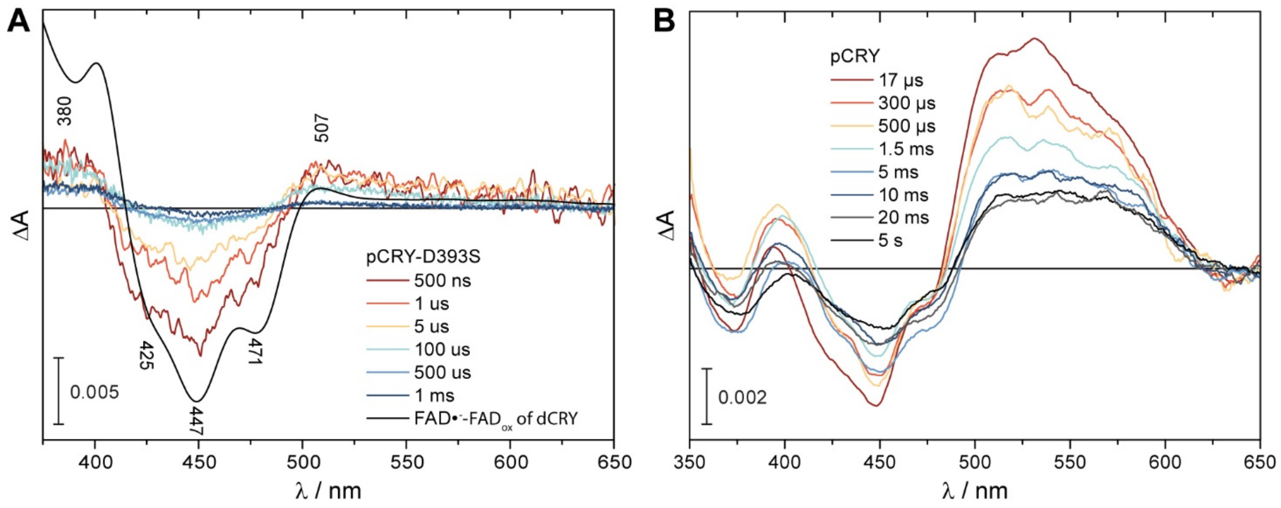

Figure S4. Comparison of time-resolved UV-vis difference spectra of pCRY and the D393S mutant. (A) Illumination of pCRY-D393S leads to the formation of the anion radical, which decays within 1 ms. **(B)** In contrast to the mutant, illumination of wild-type pCRY results in the formation of a stable FAD neutral radical with a characteristic broad absorbance between 500-600 nm. Spectra of wild-type pCRY were taken from Thöing et al, 2015 (15).

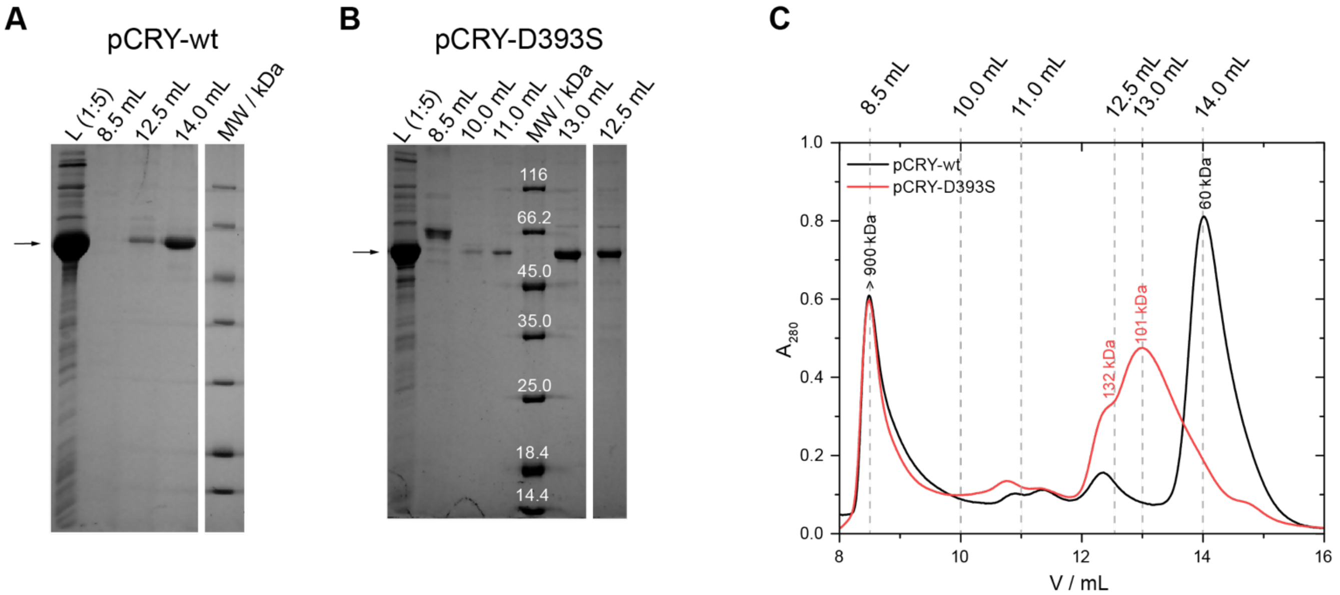

Figure S5. Analysis of elution fractions of the size exclusion chromatography via SDS-PAGE. pCRY **(A)** and D393S **(B)** samples were investigated by analytical size exclusion chromatography (SEC). SDS-PAGE analysis was performed of samples before loading on the SEC column (L(1:5); diluted 1:5) and of the elution fractions (0.5 mL) of the SEC (Fig. 3C) as indicated by their elution volume. The black arrows mark D393S and pCRY, respectively, at an apparent molecular weight of ~60 kDa and a theoretical molecular weight of 59 kDa. **(C)** The corresponding elution fractions from the SEC are indicated with dashed lines.

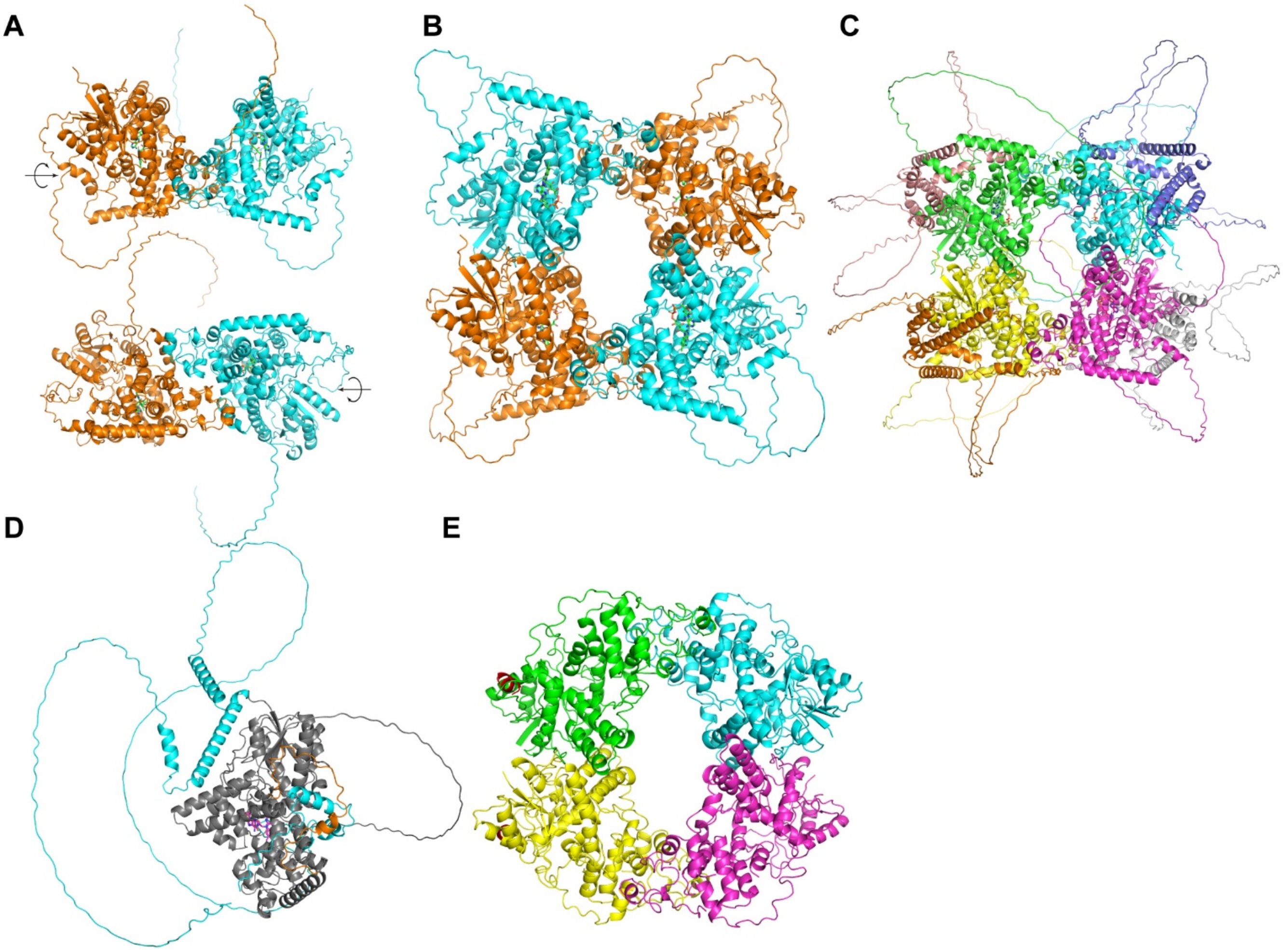

Figure S6. AlphaFold3 models of full-length CRY2 as monomer, dimer and tetramer to show the competition of CCE with CIB1, and Cryo-EM structure of tetrameric CRY2-W374A with bound CIB1 fragment. The AlphaFold3 models of CRY2 dimer **(A)** and tetramer **(B)** show two binding sites for oligomerization at the PHR domain and unstructured CCE. Binding of CIB1 competes with binding of CCE to PHR as presented in the tetramer (CIB1: orange, gray, blue and blush, respectively) **(C)** and in the monomer (CIB1: cyan; CCE: orange) **(D)**. Prediction of the CIB1 binding site by AlphaFold3 is in good agreement with the Cryo-EM structure **(E)** of tetrameric CRY2 mutant W374A with bound CIB1 fragment (red) (PDB: 7X0Y). A detailed presentation of the competition of CIB1 and CCE is shown in Fig. 4A.

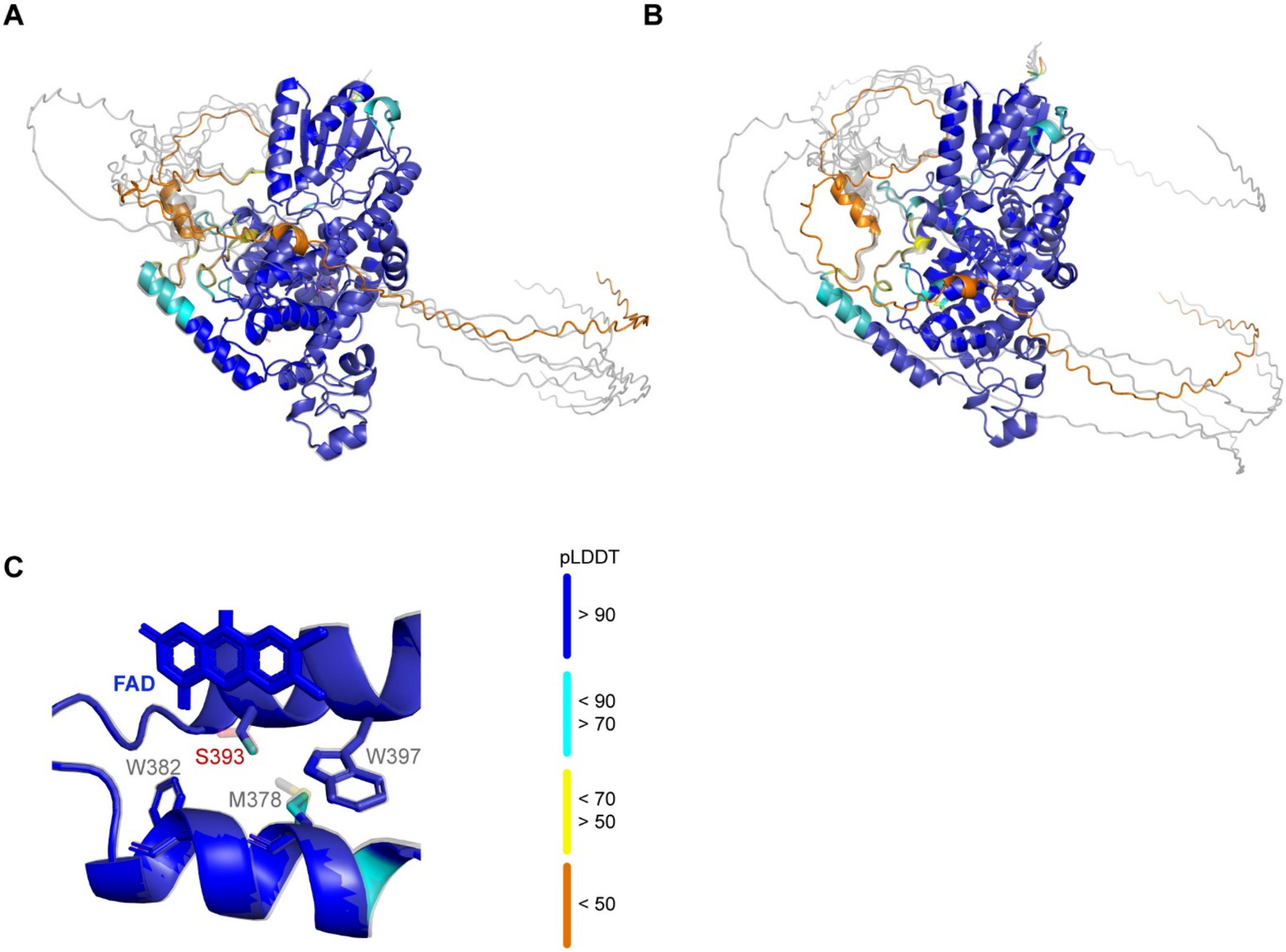

Figure S7. Five best models and pLDDT-values for CRY2 and D393S generated with AlphaFold3 and shown in Figures 4 and 5. Five best models are depicted of which the prediction confidence of the single best model is indicated by the predicted local distance difference test (pLDDT), a score that scales from 0-100 with scores >90 indicative of the highest confidence predictions. The four other predicted models are shown as an overlay in gray. **(A)** All five models of CRY2 suggest a similar interaction site of CCE with the PHR. **(B)** The overall structure of the D393S mutant is shown. **(C)** pLDDT values for atoms of D393S are shown. In all five models, hydrogen bonding to W397 and backbone of M378 is disrupted by the point mutation to serine.

Mutation	Forward oligo	Reverse oligo
V367M	TGATTGTTTCAAGCTTTGCTATG AAGTTTCTTCTCCTTCCATG	CATGGAAGGAGAAGAAACTTCAT AGCAAAGCTTGAAACAATCA
K368E	GTTTCAAGCTTTGCTGTGGAG TTTCTTCTCCTTCC	GGAAGGAGAAGAAACTC CACAGCAAAGCTTGAAAC
W374A	GTTTCTTCTCCTTCCAGCCAAATGGGGAATGAAGT	ACTTCATTCCCCATTTGGCTGGAAGGAGAAGAAAC
K375R	GAAGTTTCTTCTCCTTCCATGGAGA TGGGGAATGAAGTATTTCTGGG	CCCAGAAATACTTCATTCCCCATCT CCATGGAAGGAGAAGAAACTTC
M378R	TTCCATGGAAATGGGGAGGGAAGTATTTCTGGGA	TCCCAGAAATACTTCCCTCCCCATTTCCATGGAA
L385V	ATGAAGTATTTCTGGGATACAGTT TTGGATGCTGATTTGGAA	TTCCAAATCAGCATCCAAAAC TGTATCCCAGGAATACTTCAT
D393S	GCTGATTTGGAATGTAGTATCCTTGGCTGGC	GCCAGCCAAGGATACTACATTCCAAATCAGC
D393A	GCTGATTTGGAATGTGCT ATCCTTGGCTGGC	GCCAGCCAAGGATAGCACATTCCAAATCAGC
L395Q	TTGGAATGTGACATCCAAGGCTGGCAGTATATCTC	GAGATATACTGCCAGCCTTGGATGTCACATTCCAA
Y399S	GACATCCTTGGCTGGCAGAGT ATCTCTGGGAGTATCC	GGATACTCCCAGAGATACT CTGCCAGCCAAGGATGTC
S401F	CTTGGCTGGCAGTATATCTTT GGGAGTATCCCCGATGGC	GCCATCGGGGATACTCCCAAA GATATACTGCCAGCCAAG
P405L	GTATATCTCTGGGAGTATCCTG GATGGCCACGAGCTTG	CAAGCTCGTGGCCATCCAG GATACTCCCAGAGATATAC

Figure S8. Primers used for CRY2 mutagenesis. Shown are oligos used for generating mutations in CRY2 used in study. Mutated regions are indicated in red.

Supplementary Video Legends

Supplementary Video 1. Representative HEK293T cells expressing wild-type (WT) or indicated variants of CRY2PHR-mCherry, coexpressed with plasma membrane-localized CIBN-pmEGFP. Cells were exposed to blue light at the start of imaging (and every 15s) then tracked for 60 s to monitor recruitment of CRY2PHR-mCherry to the plasma membrane.

Supplementary Video 2. Representative HEK293T cells expressing wild-type (WT) or indicated CRY2PHR-EYFP variants, as relates to Figure 2D&E. Cells were exposed to blue light at the start of the imaging (and every 5s), then tracked for 90 s to monitor clustering in response to blue light exposure.
